# Effects of Treatment with Liraglutide Early after Surgical Intervention on Clinical Outcomes in Patients with Short Bowel Syndrome: A Pilot Observational “Real-Life” Study

**DOI:** 10.3390/nu15122740

**Published:** 2023-06-14

**Authors:** Fabio Dario Merlo, Umberto Aimasso, Marta Ossola, Mirko Ippolito, Leila Cravero, Valentina Ponzo, Simona Bo

**Affiliations:** 1 Dietetic and Clinical Nutrition Unit, Città della Salute e della Scienza Hospital, C.so Bramante 88, 10126 Torino, Italy; fdmerlo@cittadellasalute.to.it (F.D.M.); uaimasso@cittadellasalute.to.it (U.A.); mossola@cittadellasalute.to.it (M.O.); mippolito@cittadellasalute.to.it (M.I.); leila.cravero@unito.it (L.C.); 2 Department of Medical Science, University of Torino, C.so Dogliotti 14, 10126 Torino, Italy; valentina.ponzo@unito.it

**Keywords:** fecal loss, glucagon-like peptide-1 agonist, liraglutide, ostomy output, short bowel syndrome

## Abstract

Liraglutide, a glucagon-like peptide-1 agonist, has been shown to have beneficial effects on fecal output in short bowel syndrome (SBS) by small human studies. Its potential effects early after gut resection are not known. In this pilot observational study, we described the 1- and 6-month liraglutide effects in 19 adult patients with a new SBS diagnosis within 1 month after surgical resection. Stomal/fecal and urinary outcomes, serum/urinary electrolytes, and body composition were assessed. Both within-group differences and between-group comparisons with 20 SBS patients refusing liraglutide treatment were evaluated. The main liraglutide-related side effect was mild nausea, except in one patient, who experienced severe nausea/vomiting. The median ostomy/fecal output was significantly reduced by −550 mL/day after 6 months of treatment (*vs*. −200 mL/day in untreated, *p* = 0.04). The number of patients reaching a ≥20% output reduction was 10/19 (52.6%) treated *vs*. 3/20 (15.0%) untreated patients (*p* = 0.013) at 1 month and 12/19 (63.2%) *vs*. 6/20 (30.0%) (*p* = 0.038) at 6 months, respectively. Participants with a clinically relevant output reduction at 6 months had a significantly lower baseline weight and BMI. Energy parenteral supply significantly decreased, while infused volumes, oral energy, and fluid intakes slightly decreased, though not significantly. This pilot study supports liraglutide benefits in ostomy/fecal output early after surgical gut resection in SBS patients, particularly in those with lower baseline weight values.

## 1. Introduction

Chronic intestinal failure (CIF) is the long-lasting reduction of gut function below the minimum necessary for nutrients and/or water/electrolytes absorption, requiring intravenous supplementation to maintain body health and function, and nutritional homeostasis [1].

Short bowel syndrome (SBS) is the most common cause underlying CIF and derives from extensive surgical resection due to various diseases, such as ischemic, neoplastic, inflammatory, or congenital bowel diseases [2]. Patients with SBS develop a reduced absorptive capacity with high fluid loss from ostomy due to gastric and intestinal hypersecretion, reduced area of mucosal surface, and loss of the ileocecal sphincter and accelerated gastrointestinal motility because of impaired jejunal–ileal brake, i.e., the slowing of transit in the proximal and distal small intestine which facilitates digestive and absorptive processes after food consumption [2,3]. SBS involves deficiency of the proglucagon-derived hormones glucagon-like peptide (GLP)-1 and GLP-2 [4], which both play a relevant role in gut functions. GLP-1 delays gastric emptying, prolongs intestinal transit, and promotes satiety at the central nervous system level, while GLP-2 stimulates villus growth and enhances transepithelial absorption [5,6]. The GLP-2 agonist teduglutide was the first receptor agonist to be used in clinical practice, with strong beneficial effects on the reduction/weaning from parenteral support due to the lowering of intestinal losses because of its hypertrophic effects on bowel mucosa, the inhibition of gastric acid secretions, anti-inflammatory effects, and delayed gastric emptying [7]. Both GLP-1 and GLP-2 are secreted from L cells in equimolar amounts, and, therefore, GLP-1 deficiency may also play a role in the pathophysiology of CIF.

Following extensive gut resection, depending on the extent and location of the resection, the absorptive capacity and length of the remnant bowel, the underlying disease, and the dysfunction in the remaining intestine, patients experience malabsorption of fluid, energy, and nutrients [8]. Based on individual requirements, administration of intravenous fluid and nutrients is required, often on a daily schedule [8]. Early on after surgery, antisecretory and antidiarrheal medication are the available drugs, since GLP-2 agonists can be reimbursed, at least in Italy, at 6 months after surgery, with the only exception being cases with a very short residual small bowel (<75 cm) in type 1 SBS [9]. Finding a treatment that is potentially effective in reducing fecal output early in the postoperative period and is not affected by the intestinal absorption capacity is therefore a challenge.

Contrary to the large number of studies on GLP-2, very few and small human studies have assessed the effects of GLP-1 agonists on ostomy output in SBS patients [10,11,12]. Overall, a benefit to bowel movement, diarrhea, and fecal excretions has been documented, but this effect was shown to be less pronounced than with GLP-2 agonists. In particular, exenatide ameliorated the dysregulated antro-duodenal motility of five patients with SBS [10]. Liraglutide reduced ostomy output and increased urine production in eight end-jejunostomy patients [11]. The combined acute continuous infusion of native GLP-1 and GLP-2 to nine SBS patients had additive positive effects on sodium and water as well as on energy absorption compared with either peptide alone [12]. The follow-up time of these studies ranged between 72 h [12] and 8 weeks [11], while it was only approximately reported in the case series paper [10]. Finally, previous studies enrolled chronic patients on PN for at least 6 months [10,11]. No data are at present available about the effects of GLP-1 agonists at an earlier phase of the disease, i.e., within the first month after surgery.

In the present open-label pilot observational study, we aimed to describe the 1- and 6-month effects of liraglutide on many clinical outcomes in SBS patients early after surgical intervention. Comparisons with an untreated group of SBS patients were performed too.

## 2. Materials and Methods

This was a single-center pilot observational study. All the adult patients with a new diagnosis of SBS after surgical gut resection requiring parenteral nutrition (PN) and followed up at the Intestinal Failure Unit of the “Città della Salute e della Scienza” Hospital of Torino, a tertiary referral center for CIF support, from 1 January 2017 to 30 June 2022 were evaluated for suitability for liraglutide therapy.

The inclusion criteria were age ≥18 years, surgical gut resection within 1 month, new diagnosis of SBS confirmed by radiological examination, need for PN, and ongoing treatment with antisecretory agents (pantoprazole or lansoprazole) and antidiarrheal medication (loperamide) for at least 3 weeks at the maximum dosage tolerated by the patients. Exclusion criteria were active neoplastic disease and/or being under antineoplastic treatment within the previous 5 years, inability to give informed consent, critically ill patients or patients with a <6-month life expectancy, presence of renal, liver, or cardiac diseases, or contraindications to GLP-1 agonists (i.e., hypersensitivity to the drugs, acute pancreatitis). These criteria were formulated to include a relatively homogeneous group of patients and to exclude conditions potentially interfering with fecal output, fluid and electrolyte balance, life expectancy, and the possibility to perform an adequate follow up of the patients. Out of 59 patients with a new diagnosis of SBS, 46 satisfied the above-reported criteria. Of them, 22 accepted liraglutide treatment, and 24 refused the injections.

Information on untreated patients was obtained from the database of the Intestinal Failure Unit to compare ostomy output and fecal losses to those of liraglutide-treated participants. Complete data were available for 20 out of 24 untreated patients and were collected at comparable times to those of the liraglutide-treated patients, i.e., within 1 month after surgical resection (enrolment) and 1 and 6 months after enrolment (Supplementary Figure S1).

### 2.1. Ethical Aspects

All patients gave their informed written consent to the collection and processing of their data. In addition, patients signed a form accepting the off-label administration of liraglutide. This module explained in detail the potential risks and adverse events related to the administration of the drug. The study was approved by the local ethics committee (protocol number 595/2022); all the procedures were in line with the principles of the Declaration of Helsinki.

### 2.2. Outcomes

The primary outcome was the assessment of the ostomy output and fecal losses of the patients at 1 and 6 months after liraglutide treatment. The secondary outcomes were changes in body weight and serum and urinary electrolytes, number of nights/week of PN, and volume of the infused solutions. Finally, fecal outputs of liraglutide-treated patients were compared with those of the untreated group.

### 2.3. Data Collection

The Intestinal Failure Unit database contains information about all patients with CIF since 1985 [13]. Patients were followed up from their diagnosis of CIF until weaning from PN or death. All laboratory and radiological examinations, anthropometric values, and clinical data were properly recorded.

Demographic, clinical, and laboratory data were extracted from the database.

### 2.4. Procedures

All patients received nutritional support according to their individualized needs. Personalized daily fluid and nutritional parenteral programs were balanced according to nutritional requirements, considering body weight, 24 h urine and ostomy output, oral caloric intakes, and laboratory exams in line with a standardized procedure by the same two trained physicians.

PN was administered by an intermittent schedule at night for 10–16 h/day. All patients were given a diet restricted in fiber and lactose with an energy amount appropriate for the individual needs and corrected for the estimated fecal energy losses. The intake of both oral hypotonic and hypertonic fluids was restricted. Nutritional care, centralized laboratory and radiological exams, and handling of central venous catheters and of PN complications were performed in line with recommendations [1].

Specialized nurses visited patients at their home monthly (or as needed) to ensure adequate home care support. Patients were contacted by telephone every 15 days by the physicians of the Intestinal Failure Unit, while hospital follow-up visits were performed every 30 days.

Body weight and height were measured, with the patients wearing light clothes and no shoes, by a mechanical column scale (SECA model 711) and a stadiometer (SECA 220 measuring rod, Hamburg, Germany) after fasting in the morning, after urination, and after emptying stoma bags or defecation. Body composition was determined by single-frequency bioelectrical impedance using the manufacturer’s equations (BIA 101, Akern, Florence, Italy). The phase angle (measured in degrees) was obtained by bioelectrical analysis and is an indicator reflecting cell physiological functional level and volume. Arterial blood pressure values were measured on the left arm, while sitting, after at least 10 min of rest, with a mercury sphygmomanometer with appropriate cuff sizes (ERKA Perfect-Aneroid, Bad Tölz, Germany). Blood and urinary samples were collected after an overnight fast. The laboratory methods were centrally measured according to standardized methods. Energy intakes were assessed during each visit by the same trained dietician by asking the frequency and portions of all the foods consumed in the last 7 days before the visit.

Liraglutide (Victoza^®^; Novo Nordisk, Bagsværd, Denmark; ready-to-use 6 mg/mL prefilled pens) was subcutaneously self-injected by the patients in the abdomen or thigh every morning. Starting dose was 0.6 mg/day for 1 week, followed by 1.2 mg/day for another week and then 1.8 mg/day until the end of the observation period. The dose schedule could be modified based on the individual tolerance to consist of a slower dose escalation (every 2 weeks) or maintaining the maximum tolerated dose. During the follow up, the dose of liraglutide could be changed according to the clinical response. Compliance to liraglutide treatment was assessed during each telephone call and visit.

### 2.5. Definitions

The same trained radiologist performed a barium or Gastrografin follow-through examination to estimate the residual intestinal length in all patients. In our unit, the radiological evaluation of the intestinal length was found to be more precise than the surgical measurement. Colon length, in the case of colon in continuity, was described according to the method of Cummings [14]. SBS was diagnosed in the presence of a remnant small bowel length ≤200 cm [1]. Patients with SBS were divided into the following categories according to their anatomic characteristics: type 1 (end-jejunostomy), type 2 (jejunocolonic anastomosis), type 3 (jejunoileal anastomosis). No patient underwent intestinal transplantation or reconstructive surgery, i.e., stoma closure and restoration of intestinal continuity, during the follow-up period.

A ≥20% reduction in the ostomy output of fecal losses from baseline values at 6 months was considered as clinically relevant and defined patients as “responders” to liraglutide. Weaning from PN was defined when the complete discontinuation of the treatment with the maintenance of the patient clinical stability (i.e., the maintenance of an adequate urine output, stable body weight, and values within range of serum and urinary electrolyte values for at least 6 months) occurred [10].

### 2.6. Statistical Analyses

Results are reported as mean ± SD or percentages. Within-pair differences were tested by one-way repeated measures ANOVA or, in the case of non-normally distributed values, by Friedman ANOVA. Variable values at 1 month and 6 months were compared to baseline values by paired *t*-test or Wilcoxon matched-pairs test, as appropriate. Between-group differences were analyzed by Student’s *t*-test or the Mann–Whitney test. A multiple logistic regression was employed to evaluate the association between liraglutide response and clinical variables.

## 3. Results

Out of the 22 enrolled participants, one patient experienced severe vomiting and nausea, leading to the early discontinuation of liraglutide; two other patients suspended the drug between the third and sixth months due to unwillingness to continue subcutaneous injections. The data of 19 patients were therefore analyzed.

Most participants were middle-aged males with end-jejunostomy (Table 1). The underlying diseases of the patients were more frequently surgical complications, inflammatory bowel disease, or mesenteric ischemia.

All patients started PN after the surgical resection and were on the maximum tolerated doses of antisecretory and antidiarrheal medication. These dosages were not changed during the 6-month period of observation.

No patient reported either hypoglycemia or serious adverse effects. During follow up, liraglutide dosage could be changed in order to achieve the best balance between maximizing therapeutical effects and minimizing side effects. The main side effect was mild nausea (with loss of appetite without significant decrease in oral intakes) reported by eight patients and requiring the reduction of the drug dosage to 1.2 mg. After this therapeutic change, the symptom resolved.

During the follow up, a slight, not significant increase in body weight, BMI, and fat-free mass, cellular mass, phase angle, and urinary 24 h sodium excretion was observed (Table 2). The urinary output did not change significantly. The ostomy/fecal output was significantly reduced after 6 months of liraglutide treatment by a median value of −550 mL/day; the median difference at 1 month was −400 mL/day.

Ten and twelve patients showed a ≥20% reduction in ostomy/fecal output at 1 month and 6 months, respectively. Both at 1 month and at 6 months, the presence of a clinically relevant change in the ostomy/fecal losses was associated with neither age, gender, small bowel or colon length, anatomic classification, nor with the presence of the ileocecal valve. Participants with a clinically relevant reduction at 6 months had a significantly lower baseline body weight (53.0 ± 10.1 kg in responders vs. 72.8 ± 22.3 kg in non-responders, *p* = 0.016) and baseline BMI (19.0 ± 2.7 kg/m^2^ in responders vs. 26.8 ± 10.1 kg/m^2^ in non-responders, *p* = 0.020). In a logistic regression model, the inverse association between response to liraglutide and baseline body weight (OR = 0.84; 95% CI 0.72–0.98, *p* = 0.034) remained statistically significant after adjusting for gender but not after adjustments for other variables. Response to liraglutide and baseline BMI were inversely associated after adjustments for age (OR = 0.50; 95% CI 0.25–0.98, *p* = 0.045).

The infused volumes slightly decreased, though not significantly. At 6 months, the number of nights/week of PN significantly reduced: five patients were weaned to ≥1 nights/week, one patient was fully weaned from PN, two to 1 night/weeks, one to 2 nights/week, and one to 4 nights/week (Table 2). The energy parenteral supply significantly decreased, while the unrestricted oral energy intake did not change significantly during the follow up (Table 2). Biochemical markers of the nutritional state, such as serum albumin, pre-albumin, and transferrin, significantly improved.

The clinical characteristics of the 20 untreated patients are reported in Table 1. Although this was not a case–control study, there were no significant differences for baseline variables between the two groups. Length of the remaining small bowel was similar (median values 105 cm vs. 100 cm in untreated and liraglutide-treated patients, respectively), while median values of fecal losses at baseline were slightly lower in these patients (2200 mL/day vs. 2500 mL/day in untreated and liraglutide-treated, respectively, *p* = 0.53 with Mann–Whitney test). The median ostomy/fecal output of these patients was 2200 mL/day at enrolment, 2000 mL/day at 1 month, and 2000 mL/day at 6 months (*p* for trend = 0.09) (Supplementary Table S1). The median reduction at 6 months and 1 month were, respectively, −200 mL/day (vs. −550 mL/day for liraglutide-treated patients, *p* = 0.04) and −150 mL/day (vs. −400 mL/day for liraglutide-treated patients, *p* = 0.44). The number of patients reaching a ≥20% reduction in ostomy/fecal output was three (15.0% vs. 52.6% of liraglutide-treated patients, *p* = 0.013) at 1 month and six (30.0% vs. 63.2% of liraglutide-treated patients, *p* = 0.038) at 6 months, respectively. No association between the ≥20% output reduction at 6 months and baseline weight or BMI was found in these patients. At 6 months, six patients were weaned to ≥1 nights/week PN (two to 1 night/weeks, three to 2 nights/week, and one to 3 nights/week), but three patients required more nights/week of PN (two requiring one additional night/week and one requiring two additional nights/week), and none was fully weaned (Supplementary Table S1) (*p* = 0.64 with respect to liraglutide-treated patients).

## 4. Discussion

Liraglutide treatment early after surgery was associated with a reduction in the ostomy output or fecal losses in patients with SBS.

GLP-1 is quickly released after a meal from the distal bowel and enhances insulin production and secretion, decreases glucagon secretion, and increases glucose uptake and glycogen synthesis in peripheral tissues, thus reducing serum glucose levels [15]; furthermore, it delays gastric emptying and increases satiety [15]. In rodents, GLP-1 exerts anti-inflammatory, proliferative, and trophic effects both in the small and large intestine [16,17,18]. In humans, GLP-1 agonists may act by stimulating ileo–colonic brake, reducing gut motility by normalization of the dysregulated antro-duodenal motility (thus increasing epithelial contact time), inhibiting gastric acid secretion, and decreasing appetite [10,19]. We found a significant median reduction by 550 mL/day of ostomy output or fecal losses at 6 months after starting liraglutide, which is in line with previous studies reporting decreased ostomy wet weight output [10,11]. The reduction, although not significantly different, in oral and parenteral fluids and calories with maintenance of diuresis and weight confirmed the decrease in fecal output of our patients after treatment with liraglutide. Reduction in fluid oral intake is particularly relevant in patients with type 1 SBS, because drinking large amounts of water or other hypotonic fluids leads to increased stomal output with further loss of electrolytes determined by the concentration gradient existing between the blood and the intestinal lumen [1].

According to the literature [11], the effect was rapid, within a few days, for most participants. However, a great between-individual variability was observed. Reduction in ostomy or fecal losses was not predicted by the small bowel or colon length, in line with another study [11]. Intriguingly, the baseline body weight of our participants was inversely associated with liraglutide response, arbitrarily defined as a reduction in output ≥20% of the baseline value. This finding was counterintuitive, since it was demonstrated that liraglutide treatment results in dose-dependent weight loss, thus exerting its beneficial effects in patients with obesity [20]. This result may have been determined by chance due to our low sample size. However, such an association was not found in the group of untreated patients. Recently, GLP-1 agonists have been proven to display anti-inflammatory and immune-modulatory properties, thus justifying their pleiotropic effects resulting in protection from the chronic complications of diabetes mellitus [21]. It could be hypothesized that the anti-inflammatory effect of liraglutide is less evident in patients with a chronic pro-inflammatory status, such as individuals who are overweight, while, in insulin-sensitive subjects, this effect could be enhanced. If confirmed by other studies, these results might allow the identification of patients more suitable for treatment by means of their baseline clinical characteristics.

We cannot exclude that the observed reduction in the ostomy/fecal losses may have been a consequence of an early innate intestinal adaptation, spontaneously occurring within the first 2–5 years after gut resection, due to the hypersecretion of the pro-adaptive distal hormones, gradually leading to a restoration of the intestinal absorption [19]. This process characterizes mostly type 3, and much less often type 2, SBS, while it is rare in type 1 SBS [19]. To overcome this potential confounding factor, however, we limited our evaluation to the first 6 months after surgery, a period during which the full steady state should not yet have been achieved [8]. Indeed, the spontaneous process of intestinal adaptation starts following intestinal resection by involving both functional and structural changes in the residual gut and behavioral modifications, such as hyperphagia [8,22,23,24]. To assess whether the observed changes were due to the drug effect and not to the natural compensatory processes occurring after intestinal resection, the liraglutide-treated patients were compared with an untreated group with similar baseline characteristics. We observed a significantly higher median reduction in fecal/stomal outputs at 6 months and increased percentages of clinically relevant changes in fecal losses in liraglutide-treated patients when compared to the untreated group, thus corroborating the hypothesis regarding there being a role for liraglutide early after surgery. The number of nights/week weaned from PN was not significantly different between groups, but, in a few cases in the untreated group, it was necessary to increase the number of days of infusion. However, the number of cases for each category was too small to draw reliable conclusions on this result. Furthermore, we failed to find an association between the anatomic SBS characteristics and ostomy/fecal losses, thus supporting the role of the liraglutide itself in the output reduction.

This was a small, open-label observational study, and, by its nature, it had many limitations. First, the number of studied patients was small; however, previous studies had a much smaller sample size. Furthermore, differently from others, we first assessed the effects of liraglutide administered early after surgery. In other studies, liraglutide was given to patients continuously dependent on PN for at least 6 months or longer [8,9]. We demonstrated that liraglutide can be administered as early as 1 month after surgery, safely, in the absence of serious side effects, and without weight loss or hypoglycemia. Even though mild adverse gastrointestinal effects, such as nausea, were reported, energy oral intake was not reduced significantly in our participants. Therefore, if these results are confirmed by randomized controlled trials, liraglutide treatment might represent a potential opportunity for treatment early after surgery, a condition for which antisecretory and antidiarrheal medications are the only available drugs at present since GLP-2 agonists are reimbursed at least 6 months after surgery [25].

The dosing schedule of liraglutide was not defined a priori, but was conditioned by the patient’s clinical response since this was a real-life study, and patients were treated according to standards of care. Nevertheless, our study took place at a tertiary referral center for CIF support, with skilled professionals and proper organizational and logistic facilities. The adjustments in PN were guided by a standardized approach, according to guidelines, based on body weight, 24 h urine and ostomy/fecal output, oral caloric intakes, and laboratory exams. Unfortunately, in our hospital, variables and exams of potential interest, such as fecal energy and macronutrients, hormone profile, tests for gastric emptying, and analysis of the histological changes in the gut mucosa, are not routinely performed. Finally, a mix of residual anatomy, including patients with at least a part of a colon in continuity, may have affected the fecal volume losses of our patients. A randomized controlled trial on a large number of participants would allow an adequate evaluation of the effects of liraglutide by overcoming the potential confounding effects of an observational study without a control group.

## 5. Conclusions

In conclusion, this small pilot observational “real-life” study supported the benefits of liraglutide in terms of ostomy/fecal output early after surgical resection in SBS patients. Unexpectedly, the greatest benefits were observed in patients with lower baseline weight values. These results are worth testing by a randomized, placebo-controlled trial with a larger sample size.

## Figures and Tables

**Table 1:** Characteristics of the enrolled patients.

	Liraglutide-Treated	Untreated
N of Patients	19	20
Age (years)	50.8 ± 16.6	56.1 ± 13.9
Males (%)	63.2	55.0
Underlying disease (%)		
Inflammatory bowel disease	21.1	25.0
Surgical complications	57.9	55.0
Mesenteric ischemia	15.8	15.0
Pseudo-obstruction	5.3	5.0
SBS (*n*, %)		
Type 1	13, 68.4	11, 55.0
Type 2	4, 21.1	5, 25.0
Type 3	2, 10.5	4, 20.0
Small bowel length (*n*, %)		
<100 cm	9, 47.4	8, 40.0
≥100 cm	10, 52.6	12, 60.0
Colon length (%)		
<50%	33.3	33.3
≥50%	66.7	66.7

**Table 2:** Changes in the anthropometric and clinical variables at 1 and 6 months after starting liraglutide treatment.

Variable	Enrolment	1 Month	6 Months	*p* for Trend ^^^
Number	19	19	19	
Weight (kg)	60.3 ± 18.0	61.3 ± 18.1	62.8 ± 16.6	0.10
BMI (kg/m^2^)	21.9 ± 7.3	22.1 ± 7.3	22.5 ± 6.8	0.37
Free-fat mass (kg)	25.6 ± 6.3	27.2 ± 8.2	27.6 ± 7.4 *	0.23
Cellular mass (kg)	20.0 ± 5.3	21.4 ± 7.1	22.2 ± 6.3 *	0.24
Phase angle (degrees)	4.6 ± 1.0	4.8 ± 1.5	4.9 ± 1.0	0.51
Stomal/fecal output (mL/day) ^†^	2500.0 (1750.0)	2000.0 (2000.0)	1500.0 (1800.0) ^§^	0.039
Urinary output (mL/day)	1450.0 ± 603.9	1678.9 ± 817.1	1365.8 ± 662.1	0.36
Urinary sodium (mmol/day) ^†^	59.0 (92.0)	66.0 (182.0)	116.0 (184.0)	0.17
Total volume infused (mL/day)	2478.3 ± 1022.7	2342.1 ± 1101.3	2166.4 ± 1308.5	0.35
Fluid, oral intake (mL/day)	2139.5 ± 835.1	2113.2 ± 958.6	1836.8 ± 705.9	0.32
Energy, parenteral supply (kcal/day)	1310.5 ± 593.7	1121.2 ± 480.9	927.3 ± 597.8 *	0.007
Number of nights/week of parenteral supply (%)				
7	84.2	68.4	63.2 ^§^	0.018
6	0	5.3	5.3	
5	15.8	21.1	15.8	
4	0	0	5.3	
3	0	5.3	5.3	
0	0	0	5.3	
Energy, oral intake (kcal/day)	1965.7 ± 1335.9	1680.0 ± 806.0	1730.8 ± 537.4	0.41
Serum albumin (g/dL)	3.2 ± 0.5	4.1 ± 0.6 **	4.0 ± 0.6 **	<0.001
Serum transferrin (mg/dL)	202.1 ± 50.0	272.5 ± 76.6 **	258.2 ± 83.8 **	<0.001
Serum pre-albumin (g/dL)	22.3 ± 7.1	29.4 ± 9.1 **	27.1 ± 5.9 *	0.004

^^^ *p* by one-way repeated measures ANOVA or by Friedman ANOVA. * *p* < 0.05 by paired *t*-test with respect to baseline values; ** *p* < 0.01 by paired *t*-test with respect to baseline values. ^†^ median (interquartile range) for non-normally distributed variables. ^§^ *p* < 0.05 by Wilcoxon matched-pairs test with respect to baseline values.

## Data Availability

The data presented in this study are available on request from the corresponding author. The data are not publicly available due to privacy restrictions.

## Supplementary Materials

The following supporting information can be downloaded at: https://www.mdpi.com/article/10.3390/nu15122740/s1, Figure S1: Enrolment of patients; Table S1: Anthropometric and clinical variables at enrolment and after 1, and 6-months after enrolment in untreated patients
