# Supplementary material for: Effects of Treatment with Liraglutide Early after Surgical Intervention on Clinical Outcomes in Patients with Short Bowel Syndrome: A Pilot Observational “Real-Life” Study
Source: Nutrients. 2023 Jun 14;15(12):2740. doi: 10.3390/nu15122740 (PMC10305110; doi:10.3390/nu15122740)
Supplement: Supplementary file 1 [file nutrients-15-02740-s001.zip › nutrients-2419634-supplementary.pdf]

Supplementary Table S1 Anthropometric and clinical variables at enrolment and after 1, and 6-months after enrolment in untreated patients.

**Variable**	**Enrolment**	**1-month**	**6-month**	**p-for trend^**
Number	20	20	20	
Weight (kg)	61.4 ± 16.2	62.1 ± 16.7	61.7 ± 15.4	0.06
BMI (kg/m^2)	22.4 ± 4.9	22.7 ± 5.3	22.5 ± 4.8	0.06
Free-fat mass (kg)	26.7 ± 6.7	27.2 ± 9.4	27.9 ± 10.2	0.70
Cellular mass (kg)	21.2 ± 5.4	21.8 ± 7.7	21.4 ± 7.8	0.95
Phase angle (degrees)	5.1 ± 0.8	4.8 ± 0.6	4.8 ± 1.0	0.06
Stomal/fecal output (mL/day)†	2200.0 (550.0)	2000.0 (500.0)	2000.0 (550.0)	0.09
Urinary output (mL/day)	1355.0 ± 232.8	1455.0 ± 494.2	1337.9 ± 488.9	0.52
Urinary sodium (mmol/day)†	64.5 (110.0)	82.0 (140.3)	87.0 (84.5)	0.18
Total volume infused (mL/day)	2116.0 ± 973.0	2058.1 ± 856.7	1772.5 ± 669.6	0.10
Fluid, oral intake (mL/day)	2037.5 ± 713.9	1745.0 ± 785.5	1777.5 ± 830.3	0.89
Energy, parenteral supply (kcal/day)	1043.0 ± 519.0	1044.0 ± 519.6	864.4 ± 529.3	0.020
Number of nights/week of parenteral supply (%)				
7	75.0	70.0	70.0	0.60
6	15.0	15.0	0	
5	10.0	15.0	20.0	
4	0	0	10.0	
3	0	0	0	
0	0	0	0	
Energy, oral intake (kcal/day)	1702.5 ± 523.0	1617.0 ± 402.0	1639.5 ± 502.2	0.89
Serum albumin (g/dL)	3.4 ± 0.6	4.0 ± 0.4*	3.9 ± 0.5*	0.007
Serum transferrin (mg/dL)	232.2 ± 94.4	265.1 ± 74.1	263.2 ± 72.6	0.12
Serum pre-albumin (g/dL)	23.5 ± 6.4	26.1 ± 6.9	24.7 ± 7.6	0.39

^ p by one-way repeated measures ANOVA or by Friedman ANOVA
* p < 0.01 by paired t-test with respect to baseline values
† median (interquartile range) for non-normally distributed variables

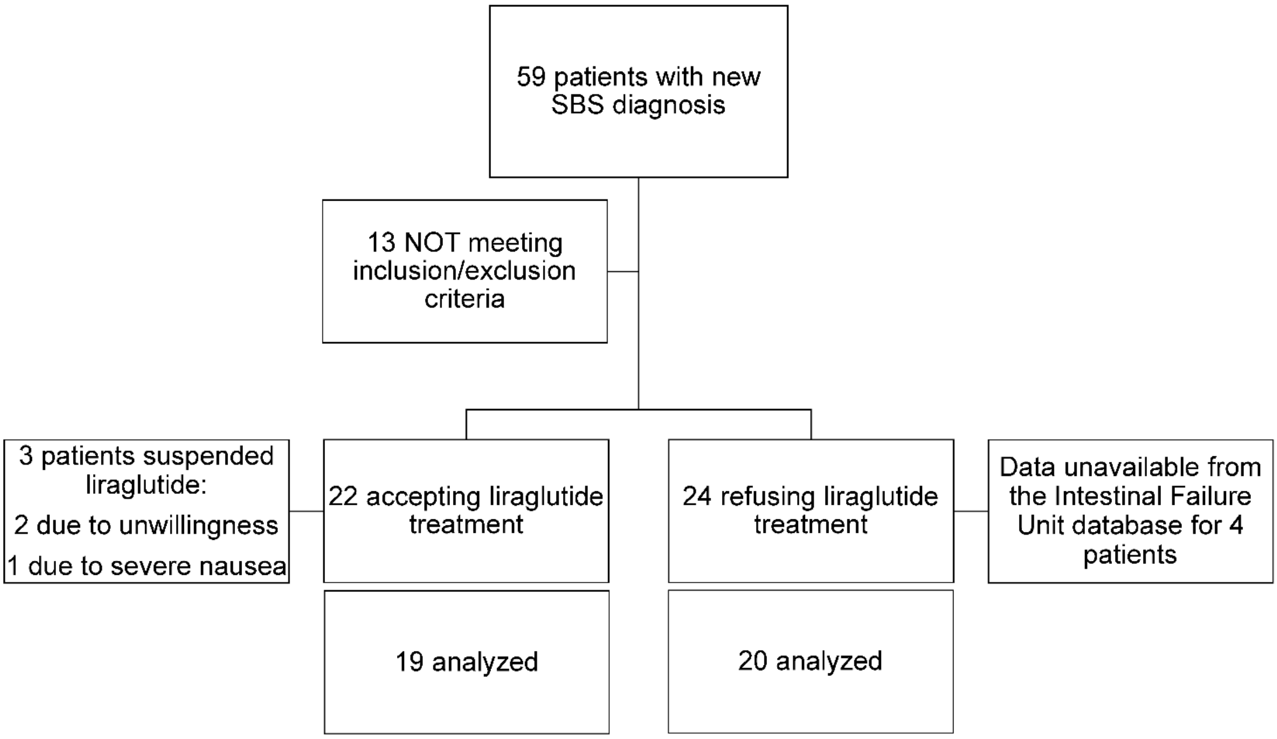

Figure S1: Enrolment of patients.
